# High Frequency of Osteophytes Detected by High-Resolution Ultrasound at the Finger Joints of Asymptomatic Factory Workers

**DOI:** 10.3390/jpm13091343

**Published:** 2023-08-30

**Authors:** Mario Giulini, Ralph Brinks, Stefan Vordenbäumen, Hasan Acar, Jutta G. Richter, Xenofon Baraliakos, Benedikt Ostendorf, Matthias Schneider, Oliver Sander, Philipp Sewerin

**Affiliations:** 1 Department and Hiller-Research-Unit for Rheumatology, Medical Faculty, Heinrich-Heine-University Düsseldorf, Moorenstr. 5, 40225 Düsseldorf, Germany; 2 Rheumazentrum Ruhrgebiet, Ruhr University Bochum, Claudiusstrasse 45, 44649 Herne, Germany

**Keywords:** osteoarthritis, screening, prevalence, ultrasound, imaging, osteophytes

## Abstract

Hand Osteoarthritis (HOA) is a frequently occurring musculoskeletal disease that impacts health. Diagnostic criteria often incorporate osteophytes documented through imaging procedures. Radiographic imaging is considered the gold standard; however, more sensitive and safer methods like ultrasound imaging are becoming increasingly important. We conducted a population-based cross-sectional study to examine the prevalence, grade, and pattern of osteophytes using high-resolution ultrasound investigation. Factory workers were recruited on-site for the study. Each participant had 26 finger joints examined using ultrasonography to grade the occurrence of osteophytes on a semi-quantitative scale ranging from 0–3, where higher scores indicate larger osteophytes. A total of 427 participants (mean age 53.5 years, range 20–79 years) were included, resulting in 11,000 joints scored. At least one osteophyte was found in 4546 out of 11,000 (41.3%) joints or in 426 out of 427 (99.8%) participants, but only 5.0% (553) of the joints showed grade 2 or 3 osteophytes. The total osteophyte sum score increased by 0.18 per year as age increased (*p* < 0.001). The distal interphalangeal joints were the most commonly affected, with 61%, followed by the proximal interphalangeal joints with 48%, carpometacarpal joint 1 with 39%, and metacarpophalangeal joints with 16%. There was no observed impact of gender or workload. In conclusion, ultrasound imaging proves to be a practical screening tool for osteophytes and HOA. Grade 1 osteophytes are often detected in the working population through ultrasound assessments and their incidence increases with age. The occurrence of grade 2 or 3 osteophytes is less frequent and indicates the clinical presence of HOA. Subsequent evaluations are imperative to ascertain the predictive significance of early osteophytes.

## 1. Introduction

Hand osteoarthritis (HOA) is one of the most common musculoskeletal disorders affecting the hand, predominantly in older women [1,2]. The clinical spectrum ranges from less symptomatic to severe impairment of quality of life [3]. Imaging techniques are crucial when clinical symptoms are suspicious for osteoarthritis. However, imaging interpretation is not always straightforward: the prevalence of HOA varies widely depending on the modality used for assessment, such as clinical examination, conventional radiography, magnetic resonance imaging (MRI), or ultrasound. Because of the various forms and patterns of HOA, there is no single standardized procedure for diagnosing HOA.

Clinically, symptomatic HOA can be classified according to the American College of Rheumatology (ACR) clinical criteria when hard tissue enlargement is found in two or more of ten selected finger joints, including at least two DIP joints, and fewer than three MCP swellings [4].

Radiographically, the Kellgren and Lawrence criteria allow for imaging detection of HOA. These criteria have been widely used in epidemiologic studies. Scoring of HOA severity is based on an imaging atlas with a range of 0–4. A score of ≥2 according to Kellgren and Lawrence is considered HOA positive. In addition to osteophytes, joint space narrowing, sclerosis, and deformity are assessed. HOA can also be detected with other imaging modalities such as US or MRI [4,5,6,7]. Overall, most studies in HOA have used conventional radiography as the imaging modality of choice.

Ultrasound imaging is playing an increasing role in diagnosis and monitoring. It is widely used in daily clinical practice, not only in the detection of inflammatory joint disease. In addition to inflammatory lesions such as synovitis or tenosynovitis, structural lesions such as erosions or osteophytes are reliably detected by ultrasound [8]. When osteophytes are detected, the concomitant diagnosis of HOA is often considered, especially in the absence of potentially inflammatory diseases. Whether and how often osteophytes are detected by ultrasound imaging in asymptomatic people, especially in the working population not involved in a musculoskeletal unit, is unknown.

People younger than 30 do not have symptomatic HOA, and the main risks are for those of age over 50 years and the female sex [9]. In the German working population, hand pain in the past week was reported by fewer than 5% of those younger than 30, but by 25% of women and 10% of men older than 50 [10].

In addition to hereditary factors, work is an independent risk factor for osteoarthritis with various mechanical stresses such as pressure, traction, vibration, and high-frequency repetitive activity. These are superimposed on psychological stresses such as tension, time pressure, break times and technical aids such as ergonomic tools. Previously described physical factors that affect the hand are extreme wrist positions, holding tools or objects with a pinch grip, highly repetitive wrist, hand and finger movements, high force application with the hand, combination of the above characteristics of posture, repetition and force, computer and mouse work and exposure to cold most of the day or exposure to vibration from hand tools more than one hour per day [11].

The objectives of this study were to investigate the prevalence and pattern of osteophytes suggestive of HOA on the hands using ultrasound imaging in a cross-sectional population-based study in an industrial working population without known HOA diagnosis.

## 2. Methods

The study was carried out in accordance with the principles of the Declaration of Helsinki, International Conference of Harmonization Good Clinical Practice guidelines, and all applicable laws and regulations with written informed consent obtained from all enrolled participants. The local ethics committee at the University of Duesseldorf approved the study and all participants provided informed written consent (trial number: 4336).

### 2.1. Participants

The study is part of a screening initiative of a referral center for rheumatologic diseases in an industrial area (Rheumazentrum Rhein-Ruhr e.V. , Duesseldorf, Germany) in cooperation with a large supraregional company. Employees from various departments and occupational groups were visited at their workplaces and offered voluntary participation in a structured examination to assess their individual risk for inflammatory and non-inflammatory musculoskeletal diseases. Individuals with known HOA or other musculoskeletal conditions affecting the hand were excluded from the study.

### 2.2. General Investigations

The general structure of the screening initiative was adopted from an earlier project with a different focus on inflammatory musculoskeletal disorders [12]. We assessed age, sex, clinical examination (pain and swelling in the joints of the hands) and a questionnaire on “recording of stress during manual work processes” [13].

### 2.3. Ultrasound Imaging

**Figure 1:**
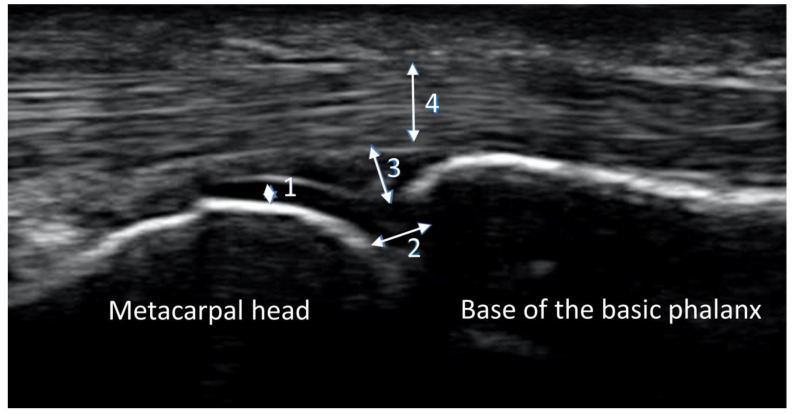
Normal MCP joint. Bone surface is regular and hyperechoic. The cartilage is homogeneously anechoic and limited cranially by a white band (1). Joint space is partly visible (2). The joint capsule is not widened (3). There is an adequate amount of synovial fluid in the capsule. The tendons (4) run across the joint and are homogeneously isoechoic.

Ultrasound imaging was performed on both hands, scanning 26 finger joints of each participant (Carpometacarpal joint CMC 1, metacarpophalangeal joints MCP 2–5, proximal interphalangeal joints PIP 2–5, and distal interphalangeal joints DIP 2–5) using an Esaote Mylab 25 Gold unit with the LA 435 linear transducer (maximum frequency 18 MHz). The seated subjects placed their hand on a small cushion in front of the examiner. Gray-scale ultrasound was performed on the palmar side with all joints in neutral position. Static images were stored and analyzed using Esaote Mylab-Desk software (version RES 1.01 ODS 13.10) to ensure standardization. Figure 1 shows an image of a normal MCP joint without major pathology.

Protrusion of the bony surface defined osteophytes. All ultrasound examinations were performed by one investigator (MG) who had completed basic and advanced ultrasound courses and had been previously trained in joint ultrasound in outpatients with musculoskeletal disorders.

### 2.4. Reading Procedures

**Figure 2:**
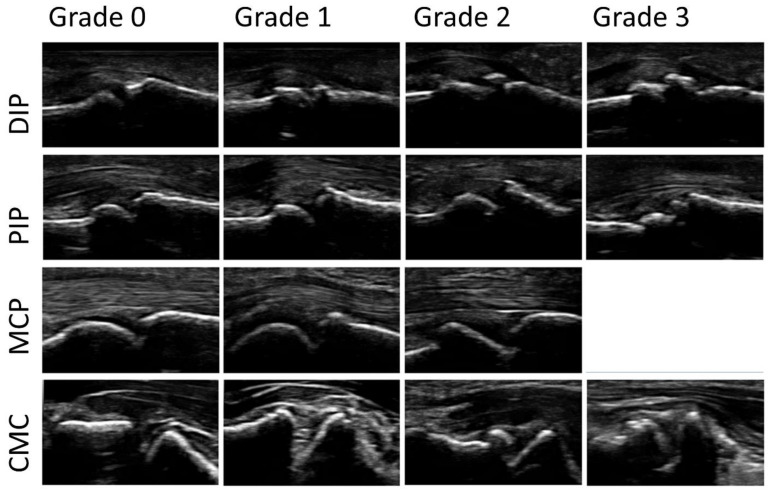
Examples of the different grades of osteophytes (0–3) for each joint group (DIP = distal interphalangeal joint; PIP = proximal interphalangeal joint; MCP = metacarpophalangeal joint; CMC = carpometacarpal joint in palmar view). No grade 3 osteophyte was found at any MCP joint.

A modified (palmar, not dorsal view) semi-quantitative score for osteophytes ranging from 0–3: 0 = no osteophytes, 1 = mild osteophytes, 2 = moderate osteophytes, and 3 = severe osteophytes was used. An increase in the score describes an increase in the severity of the osteophytes found. The largest osteophyte at each joint was scored. Figure 2 shows an example of the different grades of osteophytes for each joint group in palmar view.

The score was previously described, evaluated, and recommended by the Outcome measures in Rheumatology ultrasonography (OMERACT) group [14,15,16,17,18]. Images were graded by MG in a consensus reading with an experienced rheumatology resident (PS).

In addition, to assess the prevalence and severity of the presence of osteophytes, we calculated a sum score by adding all individual osteophyte scores of the 26 joints assessed for each participant.

### 2.5. Statistical Analysis

Descriptive analyses for continuous variables are presented as means and standard deviations. Discrete variables are presented as frequency tables and percentages. Linear regression modeling was used to examine the association between osteophyte count (dependent variable) and age and sex (independent variables). Confidence intervals for regression coefficients were calculated using direct formulas based on t values. *p* values less than 0.05 were considered significant. Calculations were performed with the statistical software R, 3.4.1 (The R Foundation for Statistical Computing).

## 3. Results

A total of 29 industrial sites in 22 cities (Bochum, Recklinghausen, Werne, Muenster, Hamburg, Lingen, Hamm, Essen, Gladbeck, Wesel, Muelheim an der Ruhr, Amsberg, Siegen, Frechen, Bergheim, Grevenbroich, Niederzier, Eschweiler, Trier, Saffig, Biblis and Grundremmingen) with participants from different working modalities were covered. The questionnaire on workload was insufficiently completed by the majority of the participants and therefore could not be used for further analysis. For the analysis, the participants were categorized as office workers and manual workers (33% vs. 67%). A total of 427 participants with a mean age of 53.5 years, ranging from 20 to 79 years (15.7% women and 84.3% men) were enrolled for the standardized ultrasound examination. While 116 images were excluded due to insufficient image quality, a total of 11,000 images of joints were evaluated. Images were available for 837 CMC1, 3386 MCP, 4239 PIP, and 3378 DIP joints.

**Figure 3:**
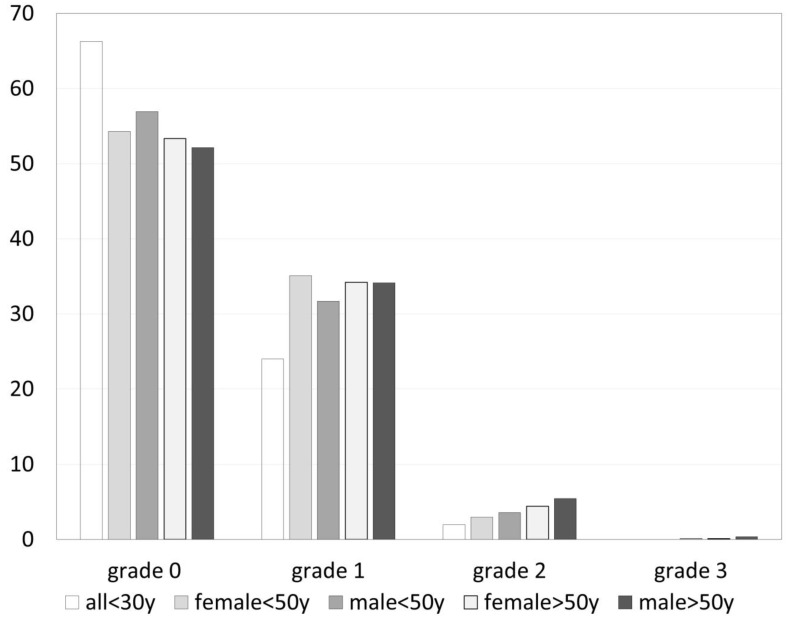
Prevalence of osteophytes (grade 0–3) related to age and gender (in percent of joints).

The prevalence of osteophytes in different joints is detailed in Table 1 with the highest patient-related prevalence in DIP joints at 61% (2057/3378), followed by PIP at 48% (1620/3399), CMC1 at 39% (325/837), and MCP joints at 16% (544/3386). Overall, DIP 3 on the right side was the most commonly involved joint, followed by DIP 3 on the left side and DIP 2 and DIP 4 on the right side and DIP 2 on the left side. The joint-related prevalence of osteophytes grade 0–3 in groups with different age and gender is compared in Figure 3.

**Figure 4:**
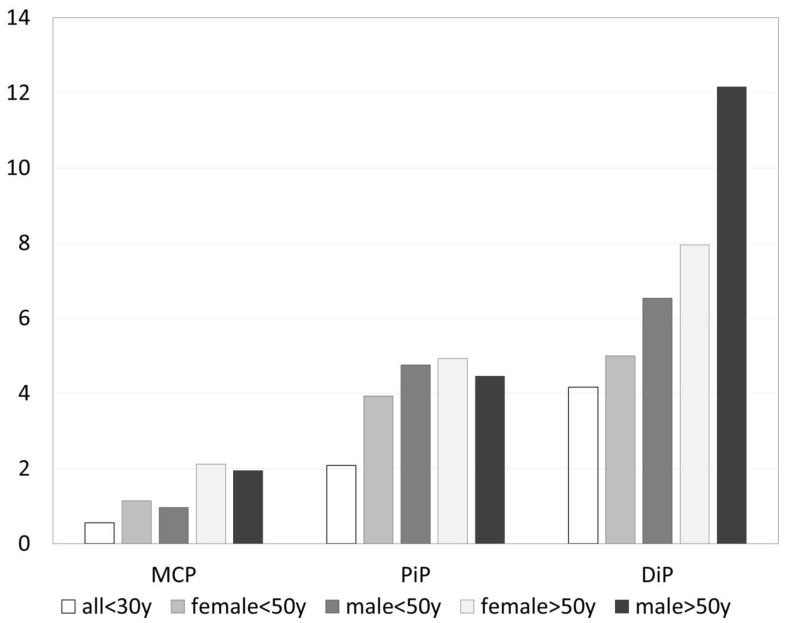
Prevalence of grade 2 osteophytes related to age and gender (in percent of MCP = metacarpophalangeal joints, PIP = proximal interphalangeal joints and DIP = distal interphalangeal joints).

Considering only grade 2 and 3 osteophytes, 553 joints were involved. Figure 4 shows the distribution of grade 2 osteophytes in MCP, PIP and DIP related to age and gender. DIP joints were the most commonly affected joint group with 9% (301/3378). The ranking was right and left DIP 3 followed by right DIP 2 and 4. CMC1 was involved in 6% (46/837), PIP in 5% (179/3399), and MCP in 1% (27/3386). MCP joints did not have any grade 3 osteophytes.

**Figure 5:**
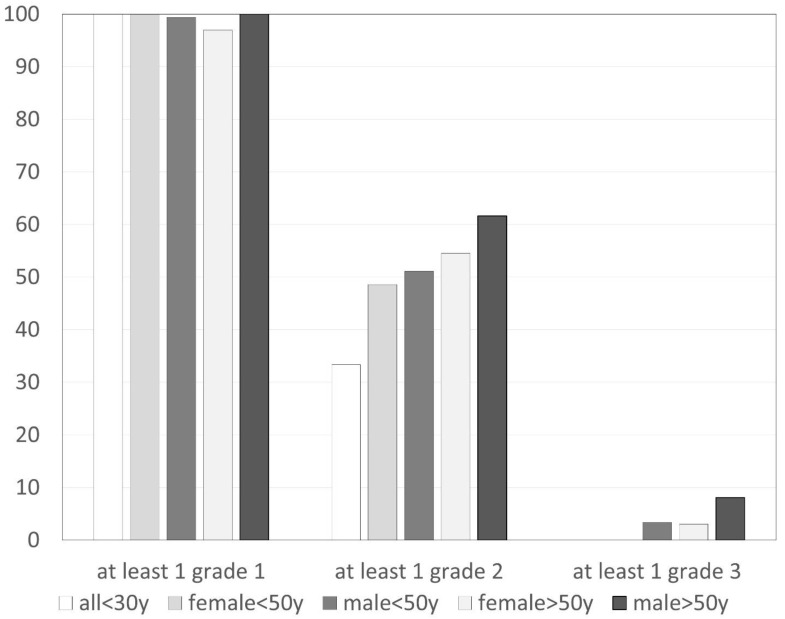
Prevalence of at least one osteophyte (grade 1–3) related to age and gender (in percent of participants).

At least one grade 1 osteophyte was found in 426/427 participants (99.8%). Exclusively grade 1 osteophytes were present in 184 (43.0%), any grade 2 in 240 (56.2%), and any grade 3 in 22 (5.2%) participants. The effect of age and gender on the prevalence of at least one osteophyte of different grades is shown in Figure 5.

**Figure 6:**
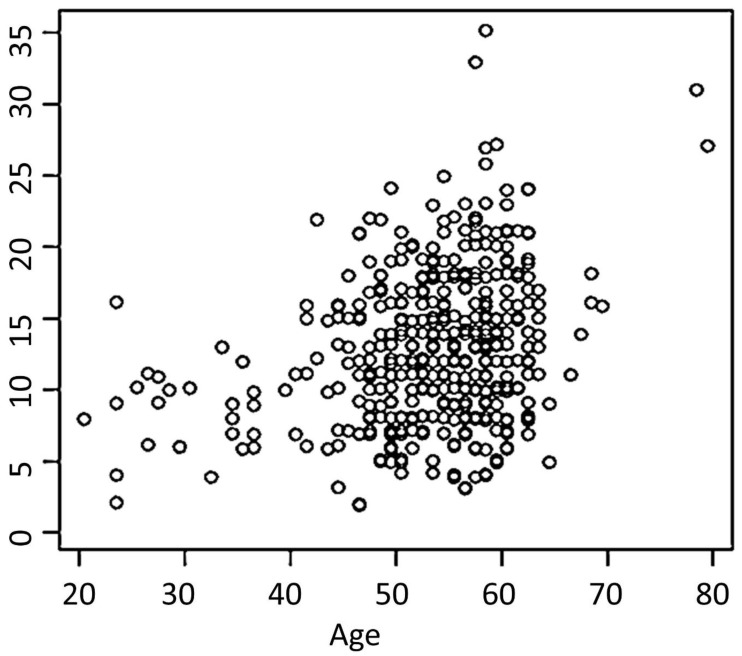
Relationship between osteophyte sum score and age. Osteophytes sum score shows summarized osteophyte grades for each subject. Osteophyte sum score increases 0.18/year (SD 0.03; *p* < 0.001).

Osteophyte sum scores increased significantly with age by 0.18/year, *p* < 0.001, Figure 6. In addition, no grade 3 osteophytes were detected in participants younger than 31 years and in females younger than 51 years. The proportion of joints without any osteophytes was higher at younger ages. The osteophyte sum score, proportion of participants and number of joints with osteophytes were not influenced by gender (*p*-value adjusted for age = 0.9). Reported workload (office vs. laborer) had no effect on the rates and patterns of osteophytes.

## 4. Discussion

The hand, with its gripping function, is essential to humans and their health. Grip strength is an independent predictor of survival or premature death, which has been confirmed by several large cohorts, although the exact relationships are still unclear [19,20,21,22,23,24,25].

Osteoarthritis of the hand is the most common disease of the hand leading to a reduction in grip strength. In the population-based NAKO cohort of 200,000 adults, 2.68% of men and 9.04% of women reported osteoarthritis of the finger joints. On clinical hand examination, 3.79% of men and 8.50% of women had pain in at least one finger joint, and 1.46% and 3.48% had more than one swollen finger joint. The frequency increases significantly after the age of 40 [9]. These findings are a motivation for research on the frequency of degenerative changes in the working population, especially with regard to early changes. Even today, osteophytes are by definition an important morphologic parameter in various imaging modalities for the confirmation of clinically suspicious HOA. It is noteworthy that most of the published studies examined participants with conventional radiology and diagnosed HOA using the Kellgren and Lawrence scoring system, making osteophytes one of the main criteria [5,26,27,28,29,30,31,32,33,34,35,36,37,38]. A systematic ultrasound evaluation of the prevalence of osteophytes in a population-based study without prior HOA diagnosis has not been performed. In our study, using ultrasound imaging, we found at least one osteophyte in all but one of the participants examined. Using conventional radiology as the primary imaging modality, studies consistently report a lower prevalence ranging from 21% to 92%, with a higher prevalence in the elderly [17,39].

The majority of joints in our cohort had no osteophytes. Most of the osteophytes we found were grade 1 osteophytes, a level that was exclusively present in 43.1% of the participants. When only grade 2 and 3 osteophytes were considered, the prevalence was lower at 56.1% and comparable to other population-based studies [26,27,28,29,30,31,33,34,35,36,37,38]. Only 5.2% of our participants had large, grade 3 osteophytes. Overall, 94.9% had mild to moderate grade 1 or 2 osteophytes.

Recently, Abraham et al. showed that, using ultrasound imaging, approximately 78% of 311 participants in the Newcastle Thousand Families Study sample had HOA signs in at least one finger joint. All participants were between 61 and 63 years of age [40]. The lower frequency of HOA reported in their study does not contradict our results, however, because only 4 joints were examined and participants were considered HOA positive if one osteophyte was found. We examined 26 joints. Thus, the likelihood of having at least one osteophyte was much higher in our study.

The most commonly used scoring system for radiographs by Kellgren and Lawrence leaves some room for interpretation: here, doubtful osteophytes are considered normal and are not scored as osteophytes [5]. As already mentioned, the resolution and therefore the sensitivity of high-frequency ultrasound exceeds that of radiographs. As a result, grade 1 osteophytes on ultrasound are classified as suspicious on conventional radiographs. According to Hart et al., small osteophytic processes in the knee should not be considered normal [41], as suggested by Mathiessen et al. in a study published in 2017 for finger joints examined by ultrasound. Both studies showed that these suspicious osteophytes can develop into larger ones [42,43]. Mathiessen et al. made these observations in previously unaffected joints of patients with HOA. It appears that osteophytes detected by ultrasound can predict the incidence of radiographic and clinically proven HOA five years later [42]. It is questionable whether these observations also apply to individuals without known HOA and whether they have an impact on the outcome.

We did not find any solitary grade 2 or grade 3 osteophytes in our study. This confirms the continuity of osteophyte growth, as osteophytes can develop from lower to higher grades even in individuals without known HOA. Based on the results of the present analysis and due to the extremely high prevalence of grade 1 osteophytes on ultrasound imaging, the occurrence of an asymptomatic grade 1 osteophyte should be considered normal. Grade 2 osteophytes allow the best discrimination for age.

Mechanical strain of the hands is a possible risk factor for HOA. There is evidence that the prevalence of osteoarthritis and HOA is etiologically related to occupation [43]. Haara et al. reported an association between workload and HOA only in women [44] and it appears that a high number of repetitive movements rather than heavy mechanical work may play a role [45]. Caspi et al. found no effect of workload on HOA expression in a cohort of patients with a relatively high mean age of 79 years [32]. Our cross-sectional study of participants from different work settings, classified as office or manual, failed to show an effect of our simplified categorization of workload on osteophyte prevalence and pattern. Unfortunately, we were not able to perform the planned detailed evaluation of hand strain because only a few participants completed the questionnaires. This is most likely due to the comparatively large amount of time required, which was not feasible due to the workplace setting. In addition, we did not assess recreational activities such as gardening or sports. We suspect an additional selection bias with a restriction to manual work in preexisting HOA. Assuming a right-handed majority in our cohort and a greater workload of the dominant hand, the higher prevalence of osteophytes on the right hand still suggests a role for mechanical factors. Only prospective and well-documented cohorts will be able to elucidate the effects of workload and behavior with certainty.

Consistent with our findings, DIP joints appear to be the most affected joints in studies using clinical examination, radiographs, and ultrasound [27,28,40,46,47]. The second most affected joint group in our cohort is PIP joints with 48% and MCP joints are barely affected. A US study by Abraham et al. estimated the prevalence in CMC 1 joints at 41%, higher than PIP joints with only 23% [40]. We evaluated more joints than other ultrasound studies and assume a higher sensitivity compared to conventional radiography. However, if only grade 2 and 3 osteophytes are considered, CMC 1 joints are also the second most commonly affected joint group in our study.

We found a significant correlation between age and an increasing number of osteophytes. Kalichman et al. described a strong correlation between osteophytes and age in all joint groups in both females and males [48]. Other studies showed similar results, but not always for every age category or joint group [27,28,32,46].

A higher prevalence of HOA in females was reported by the NAKO study [9] and Jones et al. [49]. Haugen et al. showed similar results, but the data were not significant [31], while Caspi et al. found a similar prevalence in males and females [32]. Considering that there is a sex difference in clinically apparent HOA, osteophytes seem to occur equally in males and females in our cohort. The perception of the same size of osteophyte on the more delicate female finger compared to the more robust male finger may explain this difference between clinic and imaging.

Ultrasound is an appropriate and inexpensive non-radiographic imaging modality for detecting HOA signs, but radiographs remain the gold standard of imaging modalities for diagnosing HOA. Diagnostic criteria are clinically accepted and validated [14]. Several studies have shown that ultrasound imaging is more sensitive than radiography in detecting osteophytes [16,39,50,51], synovitis [52,53,54] and erosions [50,51,55]. Furthermore, ultrasound is comparable to MRI in the detection of osteophytes in HOA [17]. Ultrasound imaging and MRI have the additional advantage of directly assessing cartilage thickness [56]. Technical advances in ultrasound devices with high image resolution and thus increased sensitivity for detecting HOA signs challenge the screening methods used to date.

The upcoming development of fast, inexpensive, and safe robotic hand ultrasound examination and interpretation by artificial intelligence will promote hand ultrasound as a population-based screening tool no longer dependent on specialists [12,57,58,59].

Thus, definitions in diagnostic criteria for HOA should recognize the increasing sensitivity for osteophytes. New scoring systems and cut-offs should be adapted to estimate the prevalence of HOA. So far, only a preliminary scoring system for HOA has been introduced. It includes osteophytes and synovitis in gray scale and power Doppler (PD) mode, if present [15]. The experts of the OMERACT group recommend the use of a semi-quantitative scoring system to detect and evaluate osteophytes using ultrasound, as in this study [18]. Further prospective observations are needed to determine the number or degree at which osteophytes in finger joints are pathological using ultrasound imaging. The present study provides values comparable to those in population-based studies using radiographs when the presence of grade 2 osteophytes is used as the ultrasound imaging criterion to estimate the prevalence of HOA.

As a limitation of our work, the interphalangeal 1 (IP1) and MCP 1 joints were not evaluated because we were unable to standardize the evaluation in a preliminary study. A dorsal view in two planes is the recommended screening for osteophytes. We chose the palmar view because, in addition to osteophytes, we evaluated synovitis, erosions, cartilage thickness, and joint space approximation during data collection. However, given our high prevalence rates, the recommendation should be re-evaluated. In addition, the voluntary nature of participation, recruitment at the workplace, and the larger male cohort may introduce bias. To minimize this bias, we approached subjects with no complaints and encouraged them to participate in the study. We graded the 11,000 images by consensus reading. A blinded double reading with a third independent reading in case of discrepancies could certainly make the results more reliable—but the feasibility is hardly possible due to the amount of work involved.

The presence of osteophytes is an important criterion for the diagnosis of HOA. The presence of osteophytes, especially low-grade osteophytes, is a normal finding in our population-based ultrasound-imaging study and may lead to overdiagnosis of HOA. The prevalence increases with age. Longitudinal observations will sharpen the boundary to clinically relevant pathology and the risk for overall progression of HOA. Despite these limitations, high sensitivity, validated grading, and automated performance will increase the relevance of hand ultrasound as a population-based screening tool in the future.

## Figures and Tables

**Table 1:** Prevalence of osteophytes in each finger joint.

	Left Hand		Right Hand	
**Joint**	**Any**	**>Grade 1**	**Any**	**>Grade 1**
**DIP 2**	59.6%	9.2%	66.7%	12.2%
**DIP 3**	67.3%	12.2%	67.4%	12.8%
**DIP 4**	56.2%	7.3%	65.6%	11.1%
**DIP 5**	47.5%	4.0%	56.8%	7.1%
**PIP 2**	45.8%	6.1%	48.5%	4.5%
**PIP 3**	53.2%	5.6%	58.7%	6.8%
**PIP 4**	51.5%	4.2%	51.4%	4.5%
**PIP 5**	34%	3.5%	38.2%	2.6%
**MCP 2**	14.6%	0.2%	19.8%	1.2%
**MCP 3**	19.7%	1.2%	28.3%	2.6%
**MCP 4**	12.3%	0.2%	18.1%	0.7%
**MCP 5**	7.3%	0.0%	8.5%	0.2%
**CMC 1**	40.1%	6.4%	37.5%	4.6%

Legend: The prevalence of any (grade 1–3) and >1 (grade 2 and 3) osteophytes in each finger joint in % for the left and right hand. DIP = distal interphalangeal joint; PIP = proximal interphalangeal joint; MCP = metacarpophalangeal joint; CMC = carpometacarpal joint.

## Data Availability

Further data could be requested from the authors.
